# The development and psychometric evaluation of COVID-19 staff questionnaire for infectious disease outbreak readiness and preparedness (SQIDORP)

**DOI:** 10.1186/s12913-022-07768-y

**Published:** 2022-03-22

**Authors:** Yangama Jokwiro, Tracy Urbanavicius, Ainsley M. Robinson, Cathy Scott, Md Rafiqul Islam

**Affiliations:** 1 Department of Nursing, Rural Health School, La Trobe University, Shepparton, Victoria 3630 Australia; 2 Goulburn Valley Health, Shepparton, Victoria Australia; 3 Department of Rural Health, The University of Melbourne, Shepparton, Victoria Australia

**Keywords:** COVID-19, Staff questionnaire, Psychometric, Reliability, Infectious disease, Health care workers

## Abstract

**Background:**

The COVID-19 pandemic has inundated the capacity of hospitals across the globe, exhausting resources, and placing extreme burden on health care workers (HCWs). Hospital preparedness during infectious disease outbreak involves development and implementation of appropriate strategies, procedures, and adequate training for HCWs. Reliable and valid tools to evaluate the perception of HCWs on the effectiveness of hospital preparedness strategies are imperative and literature is yet to fill that gap.

**Methods:**

Items for ‘The Staff Questionnaire for Infectious Disease Outbreak Readiness and Preparedness (SQIDORP)’ were selected from literature that addressed hospital preparedness during novel pandemic outbreaks. The SQIDORP was distributed within a regional hospital in Victoria, Australia. Psychometric evaluation included estimates of reliability and factor analysis while factors associated with the questionnaire were explored using regression analysis.

**Results:**

Omega coefficient of 0.89, Cronbach’s alpha coefficient of 0.88 and item-total correlations (> 0.3) indicated adequate reliability of the SQIDORP. Factor Analysis yielded three meaningful latent factors that are *effectiveness of training* (Factor 1), *self-confidence* (Factor 2) and *risk to self and stress* (Factor 3). Demographic factors did not influence the correlation with SQIDORP. However, rating ‘*the current plan for management of COVID-19 in your ward*’ and ‘*personal knowledge/skills in caring for patients with COVID-19*’ had significant positive correlation and accounted for 33% of the variance in readiness and preparedness using SQIDORP (R2 = 0.33, F = 10.227, *P* < 0.001).

**Conclusion:**

Most of the items of SQIDORP questionnaire achieved adequate internal consistence reliability. This is a valuable tool that can be utilized by hospitals to explore aspects of preparedness and give insights to the knowledge, skills, and mental health of HCWs, as perceived by the HCW themselves.

## Background

The novel Coronavirus Disease 2019 (COVID-19) outbreak, caused by Severe Acute Respiratory Syndrome Coronavirus 2 (SARS-CoV-2), originated in Wuhan, China, and rapidly advanced into a global pandemic [1]. As an international public health crisis, the COVID-19 pandemic has challenged health care, economic, and social systems worldwide; the severity of COVID-19 infection has overwhelmed hospital capacities, exhausting resources, and placing extraordinary demands on health care workers (HCWs) to provide care for a population with which they had no experience [2, 3]. Furthermore, the burden of COVID-19 has led to a rise in other infectious diseases in many countries, due to the diversion of resources to control the pandemic. This is especially challenging in low- and middle-income countries where healthcare systems are already fragile [4–7]. These factors combined have shown that adequacy of resources and staff competencies on the management of the novel disease are extremely important.

Organizational preparedness is vital when there is sudden emergence of a new infectious disease. Fundamental to organizational preparedness are skills and knowledge of the novel threat, and the management of infected patients [8, 9]. Preparedness of the hospitals includes the development and implementation of strategies for prevention, detection, and containment of the infectious disease, programs for management and support of the workforce, and procedures for response and mollification of issues that evolve from the spread of pandemics, such as shortages of *personal protective equipment*, restricted hospital capacity, and acquisition of vaccines [10, 11]. Additionally, training and education of HCWs on preparedness for a pandemic is essential to improve the experience, knowledge, skills, and mental wellbeing of staff during a pandemic [12].

While the preparation of handling a surge of patients in hospitals is central, evidence suggests monitoring the emotional and psychological burden on staff is equally essential [13–15]. Overall, HCWs exhibit higher rates of anxiety, depression, burnout, and suicidal ideation when compared to the general population [16–18]. During infectious outbreaks, there are significant psychological impacts across all population groups, with HCWs bearing a disproportionate burden [19–21]. HCWs experience multiple sources of stress during outbreak related surges, including coping with increased volume of patients, risk of nosocomial infections, fear of secondary transmission to family members, resource scarcity, stigmatisation, understaffing, and uncertainty [13, 22–24]. Additional challenges are faced by HCWs with no infectious disease expertise as they adapt to new working environments under considerable different conditions than they are accustomed with, and often with insufficient skills and training [23].

During previous infectious outbreaks, such as the Severe Acute Respiratory Syndrome (SARS) and Middle East Respiratory Syndrome (MERS) epidemics, HCWs experienced fear, anxiety, emotional distress, panic attacks, depression, psychotic symptoms, insomnia, and post-traumatic stress disorder (PTSD) [11, 13, 14, 19, 25–27]. These adverse psychological outcomes have been reported to be long lasting, persisting 6 months to 3 years post-outbreak [14, 21, 27]. Widespread anxiety, depression, stress and insomnia among HCWs was described during the early stages of the COVID-19 outbreak [28]. Further reports of the psychological impact on HCWs, including symptoms of fear, insomnia, psychological distress, burnout, and anxiety, have persistently appeared in the literature as this current global health crisis continues [15, 23, 28–32]. Like previous infectious outbreaks, the mental impact and stress induced by COVID-19 has the potential to develop into PTSD [33, 34]. Combined evidence of psychological impact on HCWs reported during SARS and MERS, and now also during COVID-19, highlights the need for continuing monitoring and support of HCWs mental health and wellbeing in infectious outbreak situations [18, 35].

Given the above, it is necessary to have a validated tool to evaluate the perception of HCWs on the effectiveness of organizational preparedness strategies from which they derive confidence to deal with the pandemic. Building confidence among HCWs is critical to combat the experience of fear and anxiety that are related to the potential of being infected during a novel pandemic [28]. These feelings of fear and anxiety are not unwarranted. During the SARS outbreak, the total number of HCW infections accounted for 21% of all confirmed cases worldwide [36], whereas in highly affected countries such as Vietnam, Canada, Philippines, France, and Singapore, HCW infections made up an even larger proportion; 28–57% of SARS cases [37]. Furthermore, 72 and 55% of all SARS cases were healthcare related in Toronto and Taiwan, respectively [38]. MERS infections among HCWs have accounted for an estimated 18% of the global total of cases [37]. In South Korea, 21% of hospital-acquired infections occurred in HCWs [39], while analyses of MERS infections in Saudi Arabia between 2016 and 2019 demonstrated that 26% of cases were in HCWs [40]. Therefore, understanding the perception of HCWs on organizational preparedness is imperative to evaluating the confidence of the workforce in dealing with the pandemic.

Given the importance of understanding how personnel may make decisions when facing competing duties, it is evident there is a paucity of adequate tools for assessing the perception of HCWs to organizational preparedness during an infectious outbreak. This is pertinent because making assumptions without adequate evidence may have serious consequences during disaster planning and management. Plans, policies, and organizational decisions should be based on the best available evidence which in turn would contribute to supporting hospital managers clinically, as well as fine tuning disaster plans for healthcare organizations. Therefore, the impetus to close the gap in literature is to have validated tools for HCWs in the preparation of a pandemic. This study provides a step towards the development of a comprehensive tool that can be used to explore aspects of preparedness, such as knowledge, skills, and mental wellbeing from the perspectives of HCWs themselves.

## Methods

### Aims

- To develop and perform the initial validation of Staff Questionnaire for Infectious Disease Outbreak Readiness and Preparedness (SQIDORP).
- To explore factors associated with the SQIDORP.

### Design, setting, and participants

A cross-sectional pilot study was conducted in a regional health service in Victoria, Australia, with 250 acute in-patient hospital beds between January and March 2020. The health service serves a catchment area of approximately one hundred and twenty thousand people, employs more than two thousand staff, and provides a range of services and programs to support the health needs of a diversity of people across all ages. This study was conducted among all levels of staff including nurses, medical doctors, allied health and other healthcare professionals.

### Study tool/ instruments: staff questionnaire

The SQIDORP scale was comprised of 19 items, formulated as statements relating to the perceived preparedness for COVID-19. Items were selected from literature that addressed organizational preparedness during novel pandemic outbreaks. Table 1 shows the items chosen for the SQIDORP and the literature supporting the selection. Responses were given on a five-point Likert-type scale ranging from (1) ‘Strongly Disagree’, (2) ‘Disagree’, (3) ‘Neutral’, (4) ‘Agree’ to (5) ‘Strongly Agree’.

**Table 1:** Items selected for Staff Questionnaire for Infectious disease outbreak preparedness (SQIDORP) and the related literature

Item	Supporting literature
1. The training provided useful information about my role and responsibilities during the COVID-19 outbreak.	[4, 5, 9]
2. The training was realistic about my role and responsibilities.	[4, 5, 9]
3. The training provided opportunity to voice my concerns.	[4, 5]
4. I have received adequate answers and support when I have voiced my concerns.	[39, 40]
5. I feel the information communicated by management is accurate and helpful.	[4, 5]
6. I am happy with the way the management are responding to COVID-19.	[4, 5]
7. I feel prepared to work on the front line with patients infected with COVID-19.	[37]
8. I feel prepared to deal with unexpected situations.	[4, 5]
9. I feel empowered to protect myself during this outbreak.	[37]
10. I am confident in my ability to provide quality care to patient with COVID-19.	[4, 5]
11. I feel well protected by management from potential infection.	[40, 41]
12. I am willing to accept the risk of this infections disease on myself and collegues.	[37]
13. I do not feel nervous and stressed about this outbreak.	[39, 40]
14. I am not likely to take time off or call in sick if I have to be on the front line.	[41]
15. I am satisfied that if I become infected, I will be able to recieve quality care.	[37]
16. My family and/or friends support my participation as a COVID-19 frontline worker.	[40]
17. I am confident in my ability to manage stress during during this outbreak.	[37]
18. I am confident that I will know how to access mental health support if needed.	[4, 5]
19. I feel the organization is very well prepared to manage COVID-19.	[4, 5]

Demographic data such as age, experience, employment status, gender, and whether they lived with a vulnerable person were included in the questionnaire. Participants were also asked to rate ‘the perceived fear of COVID-19 among patients and community is’ ‘Not at all’, ‘Slight’, ‘Neutral’, ‘Moderate’, or ‘Extreme’, to rate ‘the current plan for management of COVID-19 in their ward as’ ‘Very Unsatisfactory’, ‘Unsatisfactory’, ‘Neither’, ‘Satisfactory’, or ‘Very Satisfactory’, and their ‘knowledge/skills in caring for patients with COVID-19 as’ ‘Very Unsatisfactory’, ‘Unsatisfactory’, ‘Neither’, ‘Satisfactory’, or ‘Very Satisfactory’.

### Study procedure

The questionnaires were delivered to the wards in a box that was stored in the Nurse Unit Manager’s office, together with a sealed return box. The questionnaires were handed out to the staff during ward hand over. A participant information letter, which outlined the purpose of the study and guaranteed anonymity, accompanied each questionnaire. Consent was implied if the staff returned the questionnaire. All data were collected in March 2020. Participation in the study was completely voluntary. A total of 250 questionnaires were distributed and 179 questionnaires were completed and returned (72%).

### Statistical analysis

The Statistical Package for Social Sciences (SPSS) and AMOS, Version 24.0 (SPSS, Chicago, IL, USA) was used for statistical analysis of the data. Statistical significance was set at *P* < 0.05. Descriptive statistics were calculated, and correlations were explored using Pearson’s correlation coefficient. Internal consistency for reliability was evaluated by omega coefficient (> 0.7), Cronbach’s alpha (> 0.7), item-total correlations (> 0.3), and inter-item correlations (0.2–0.4) [41–45]. Exploratory factor analysis was used to investigate instrument dimensionality. Prior to exploratory factor analysis, Kaiser-Meyer-Olkin (KMO) measure of sampling adequacy and Bartlett’s test of sphericity were analyzed to determine the suitability of the data to undergo factor analysis; the cut offs were > 0.6 and < 1.0, and statistical significance (*P* < 0.001), respectively [46]. Linear regression was conducted using the mean score of the question as the depended variable to evaluate the factors associated with the questionnaire and the R2 change, the F statistic and *P*-values were reported.

### Ethical considerations

The study received necessary ethical approval from the Human Research Ethics Committee of the participating hospital (GVH 16/20).

## Results

### Participant characteristics

Participant characteristics are shown in Table 2. The majority of participants were aged between 21 and 40 years (60.9%), were women (86.6%), and were employed as registered and enrolled nurses (79.3%). Among the respondents, almost half of them were married (49.2%) and 31.3% were single. Approximately half of the participants had been practicing in their profession for 10 years or less (51.4%) and almost three-fifths of participants work part time (58.9%).

**Table 2:** Participant’s Characteristics (*n* = 179)

Indicators	n (%)
***Age (years)***	
21–30	69 (38.6)
31–40	40 (22.3)
41–50	29 (16.2)
51–60	21 (11.7)
61–70	5 (2.8)
Not reported	15 (8.4)
***Gender***	
Female	155 (86.6)
Male	19 (10.6)
Not reported	5 (2.8)
***Marital Status***	
Defacto	19 (10.6)
Single	56 (31.3)
Married	88 (49.2)
Other	9 (5.0)
Not reported	7 (3.9)
***Profession***	
Registered Nurse	113 (63.1)
Enrolled Nurse	29 (16.2)
Medical Doctor	9 (5.0)
Allied Health	15 (8.4)
Healthcare Assistant	4 (2.2)
Administration	6 (3.4)
Other	3 (1.7)
***Employment Status***	
Full Time	67 (37.4)
Part Time	106 (59.2)
Agency	1 (0.6)
Casual	4 (2.2)
Not reported	1 (0.6)
***Work Experience (years)***	
0–10	92 (51.4)
11–20	29 (16.2)
21–30	20 (11.2)
31–40	9 (5.0)
40+	2 (1.1)
Not reported	27 (15.1)

### Item performance

All items from the SQIDORP met the cut-off criteria for item-total correlations (> 0.3) except for item 9: ‘*I feel empowered to protect myself during this outbreak’* and item 18: *‘I am confident that I will know how to access mental health support if needed’* (Table 3). Internal consistency or reliability was indicated by a total omega coefficient of 0.89 and Cronbach’s alpha coefficient of 0.88. However, Cronbach’s alpha increased if item 9 and item 18 were deleted. The three meaningful latent factors: effectiveness of training (factor 1), self-confidence (factor 2), and risk to self and stress (factor 3) achieved a Cronbach’s alpha coefficient of 0.89, 0.86 and 0,83 respectively.

**Table 3:** Item performance of Staff Questionnaire for Infectious Disease Outbreak Readiness and Preparedness (SQIDORP)

Item	Mean	SD	Skewness	Kurtosis	Corrected Item-Total Correlation	Cronbach’s Alpha if Item Deleted
1. The training provided useful information about my role and responsibilities during the COVID-19 outbreak.	3.95	.835	−.833	.490	.628	.873
2. The training was realistic about my role and responsibilities.	3.84	.849	−.791	.356	.627	.873
3. The training provided opportunity to voice my concerns.	3.86	.878	−.641	.193	.628	.873
4. I have received adequate answers and support when I have voiced my concerns.	3.91	.894	−.726	.093	.541	.876
5. I feel the information communicated by management is accurate and helpful.	4.06	.808	−.681	.312	.632	.873
6. I am happy with the way the management are responding to COVID-19.	4.04	.869	−.657	−.155	.725	.869
7. I feel prepared to work on the front line with patients infected with COVID-19.	3.75	.894	−.288	−.607	.637	.872
8. I feel prepared to deal with unexpected situations.	3.77	.765	−.319	−.160	.443	.879
9. I feel empowered to protect myself during this outbreak.	3.16	.863	−.349	−1.599	−.353	.905
10. I am confident in my ability to provide quality care to patient with COVID-19.	3.92	.727	−.482	.283	.637	.873
11. I feel well protected by management from potential infection.	3.77	.874	−.350	−.539	.590	.874
12. I am willing to accept the risk of this infections disease on myself and collegues.	3.61	.887	−.384	−.203	.517	.877
13. I do not feel nervous and stressed about this outbreak.	3.26	.925	.213	−.808	.406	.881
14. I am not likely to take time off or call in sick if I have to be on the front line.	3.96	.817	−.579	−.091	.503	.877
15. I am satisfied that if I become infected, I will be able to recieve quality care.	4.03	.740	−.468	.076	.658	.872
16. My family and/or friends support my participation as a COVID-19 frontline worker.	4.04	.836	−.655	−.040	.392	.881
17. I am confident in my ability to manage stress during during this outbreak.	3.89	.648	−.561	1.087	.505	.877
18. I am confident that I will know how to access mental health support if needed.	3.77	.799	−.781	.533	.178	.887
19. I feel the organization is very well prepared to manage COVID-19.	3.88	.855	−.565	−.146	.755	.868

### Dimensionality

The KMO measure of sampling adequacy was 0.87 and Bartlett’s test of sphericity reached statistical significance (*P* < 0.001) which demonstrated suitability of the data for factor analysis. Subsequently, exploratory factor analysis was conducted on all 19 items in the SQIDORP. All items had correlations with at least one other item except item 9: ‘*I feel empowered to protect myself during this outbreak*’ and item 18: “*I am confident that I will know how to access mental health support if needed*” as shown in Table 3. The Kaiser criteria of an eigenvalue > 1, Cattel’s scree test, and parallel analysis yielded four latent factors which explained 60% of the total item variance. All items met the criterion of communalities exceeding 0.3 in the principal component analysis (PCA) and principal axis factoring (PAF). The 19 item SQIDORP questionnaire was primarily divided into three factors; Factor 1 with seven items (items 1–6 and 19), factor 2 with four items (items 7,8, 10 and 11), and factor 3 with six items (items 12–17) were theoretically interpreted as shown in Table 4. Additionally, item 9 and item 18 fell into a fourth factor which could not be theoretically interpreted.

**Table 4:** Factor Analysis of the Staff Questionnaire for infectious disease outbreak readiness and preparedness (SQIDORP)

Item	Factor 1: Effectiveness of training	Factor 2: Self confidence	Factor 3: Risk to self and stress	Factor 4: No theoretical fit
1. The training provided useful information about my role and responsibilities during the COVID-19 outbreak.	.841			
2. The training was realistic about my role and responsibilities.	.860			
3. The training provided opportunity to voice my concerns.	.834			
4. I have received adequate answers and support when I have voiced my concerns.	.544			
5. I feel the information communicated by management is accurate and helpful.	.538			
6. I am happy with the way the management are responding to COVID-19.	.611			
7. I feel prepared to work on the front line with patients infected with COVID-19.		.647		
8. I feel prepared to deal with unexpected situations.		.773		
9. I feel empowered to protect myself during this outbreak.				.566
10. I am confident in my ability to provide quality care to patient with COVID-19.		.569		
11. I feel well protected by management from potential infection.		.577		
12. I am willing to accept the risk of this infections disease on myself and collegues.			.678	
13. I do not feel nervous and stressed about this outbreak.			.780	
14. I am not likely to take time off or call in sick if I have to be on the front line.			.682	
15. I am satisfied that if I become infected, I will be able to recieve quality care.			.544	
16. My family and/or friends support my participation as a COVID-19 frontline worker.			.435	
17. I am confident in my ability to manage stress during during this outbreak.			.505	
18. I am confident that I will know how to access mental health support if needed.				.781
19. I feel the organization is very well prepared to manage COVID-19.	.529			

Extraction Method: Principal Component Analysis

Rotation converged in 6 iterations. Rotation Method: Varimax with Kaiser Normalization

### Factors associated with SQIDORP

Age, work experience, marital status, living with a vulnerable person, and employment status did not influence the correlation with the overall mean of the questionnaire items or mean of the above three factors of SQIDORP separately. However, rating ‘*the current plan for management of COVID-19 in your ward*’ and ‘*personal knowledge/skills in caring for patients with COVID-19*’ had significant positive correlation with the overall questionnaire mean and mean of three factors separately (*P* < 0.01; Table 5). Multivariate linear regression was conducted using the mean score of the question as the depended variable. ‘*The current plan for management of COVID-19 in your ward is*’, ‘*do you live with a vulnerable person?*’, ‘*your knowledge/skills in caring for patients with COVID-19*’ accounted for 33% of the variance in staff preparedness (R2 = 0.33, F = 10.227, *P* < 0.001).

**Table 5:** Correlations the Staff Questionnaire for infectious disease outbreak readiness and preparedness (SQIDORP) with demographic factors study question

Correlations	1	2	3	4	5	6	7	8	9	10	11	12	13	14
1. Age	1													
2. Work experience	.822^a^	1												
3. Gender	−.013	.032	1											
4. Profession	−.037	−.094	−.057	1										
5. Employment Status	.002	−.056	.262^a^	−.092	1									
6. Marital Status	.371^a^	.310^a^	−.012	−.120	−.022	1								
7. Do you live with a vulnerable person?	−.177^b^	−.209^a^	−.077	.087	.019	−.057	1							
8. The perceived fear of COVID-19 among patients and community is	.070	.060	.003	.029	.077	−.034	−.037	1						
9. The current plan for management of COVID-19 in your ward is	.078	.104	−.019	.079	.055	.031	.051	.121	1					
10. Your knowledge/skills in caring for patients with COVID-19 are	.236^a^	.251^a^	.015	−.173^b^	.083	.039	−.103	.036	.278^a^	1				
11. Factor_1	.134	.074	.002	−.011	.109	.126	−.024	−.063	.454^a^	.434^a^	1			
12. Factor_2	.119	.032	.001	−.012	.163^b^	.130	.077	.037	.362^a^	.319^a^	.625^a^	1		
13. Factor_3	−.031	−.032	.018	−.007	.098	.085	.114	−.095	.285^a^	.292^a^	.560^a^	.614^a^	1	
14. Mean questionnaire	.096	.046	−.012	−.058	.120	.128	.068	−.078	.425^a^	.414^a^	.864^a^	.874^a^	.823^a^	1

^a^Correlation is significant at the 0.01 level (2-tailed)

^b^Correlation is significant at the 0.05 level (2-tailed)

## Discussion

This study provides a novel validated tool that can be used to explore aspects of hospital pandemic preparedness, such as knowledge, skills, and mental wellbeing from the perspectives of HCWs themselves. The undertaken validation of the SQIDORP in this study demonstrated satisfactory reliability and validity estimates, and suggested factors that are consistent with available literature [47, 48]. The purpose of developing and validating the SQIDORP was to produce a tool that would enable organizations to objectively assess their pandemic plans from the perspectives of the HCWs, thereby eliminating assumptions.

Internal consistency and reliability of the SQIDORP was indicated by a total omega coefficient of 0.89, Cronbach’s alpha coefficient of 0.88 and item item-total correlations (> 0.3). Removal of item 9: *‘I feel empowered to protect myself during this outbreak’* and item 18: ‘*I am confident that I will know how to access mental health support if needed’* increased the reliability. This was an unexpected result given that the mental health toll of an infectious outbreak including symptoms of depression and PTSD among HCWs is well documented in literature [11, 13, 14, 19, 25–27]. Thus, the assumption that the perceived adequacy of physical and mental health support within the organization can influence confidence of HCWs in outbreak preparedness. It plausible to suggest that the two items did not perform well because the data was collected at the beginning of the outbreak in other countries and the risk of transmission in Australia appeared to be remote at the time. This could be explored in other countries that had significant outbreaks as literature suggests that this could be a key factor in the willingness of HCWs to work during pandemics, for which role abandonment has become a concern for policy makers [49–51].

Factor analysis yielded a solution with three meaningful latent factors: *effectiveness of training* (factor 1), *self-confidence* (factor 2), and *risk to self and stress* (factor 3) which all achieved adequate measures of reliability. This interpretation applied to readiness and preparedness in which adequate training contributes to building self-confidence and acts as a protective factor against stress and anxiety [52]. Such training should involve equipping staff with information about what to expect, how to protect themselves, how to respond to and manage infected patients, how to apply disease specific infection and prevention control measures, and how they can minimize emotional and physical stress [53, 54]. On the other hand, if training is perceived as inadequate, HCWs are more likely to feel less self-confident and to experience symptoms of burnout and PTSD, which often continue in the longer term [52]. Both risk to self and stress are key factors considered by HCWs during a pandemic. It has been well established that working in an environment where there is significant risk to self via exposure to infected patients is associated with adverse psychological outcomes which can influence HCWs’ willingness to work [11, 13, 14, 19, 25–27]. During the avian influenza outbreak in New York, only 11% of home HCWs and 37% of registered nurses were willing to work [55]. Furthermore, only 23% of community nurses in Hong Kong and 25% of HCWs in Nigeria expressed a willingness to work during the Avian influenza pandemic [56, 57]. High stress levels due to conflicting moral obligations and fear of becoming infected or transmitting the virus to family members or friends impacted the willingness of HCWs to work [55, 58]. In contrast, confidence in safety, risk perception, prior training, knowledge, and confidence in skills facilitate HCW’s willingness to work [59]. Thus, it has become vital to better understand the perception of HCWs within their context and predict how they may behave when they face a pandemic. Item 9 and item 18 contributed to a fourth latent factor which was not theoretically interpreted since reliability of the questionnaire increased when they were deleted. Given that these items did not perform well in the reliability tests, this study suggests that these items could be removed.

In this study, age was not a factor in the variability of the mean score of the questionnaire or for factor 3 (*risk to self and stress*). Conversely, it has been established that vulnerability to COVID-19 increases with advancing age [8, 9]. In addition, work experience, living with a vulnerable person, and employment status did not influence the variability of the mean score in readiness and preparedness. The difference in results may be explained by the age range of participants in this study; 60.9% of the study sample were aged between 21 and 40 years and their needs may not be the same as older HCWs. However, rating ‘*the current plan for management of COVID-19 in your ward is’* and personal *‘knowledge/skills in caring for patients with COVID-19’* accounted for 33% of the variance in staff preparedness. Key components of staff readiness and preparedness are education, training, and simulated plans [53]. These provide skills and knowledge of the novel threat and guidelines for the management of infected patients [8, 9]. Therefore, it is plausible to speculate that the widespread fear, staff depression, and high levels of stress in the early onset of the COVID-19 outbreak may have been mitigated by the confidence of HCWs in their own knowledge and skills, as well as in their organization’s planning.

### Limitations of the study

The questionnaire was trialed at the beginning of the pandemic when the extent and the knowledge of the impact was only emerging. The uniqueness of the context, that is the COVID-19 pandemic threat in Australia and subsequently a hospital about 150 km away from a major metropolitan city, could have impacted the results. Thus, contextual location of the data collection implies cautious interpretation of the findings, and further data from other contexts and countries is needed. Regarding the study procedures, although the data collection process was anonymous to encourage participants to be truthful, cross-sectional self-reported data needs careful interpretation due to social desirability bias. In addition, this was a pilot study, the sample was relatively small consisting mainly of female nurses and the data was collected at a single hospital, which may further limit generalizability for different genders and other professions.

## Conclusion

SQIDORP achieved adequate measures of internal consistence reliability for 17 of the items in this study. Given the tenacity of COVID-19 and the likeliness that this pandemic will persist, the SQIDORP provides a first step towards development of a reliable evaluation tool for hospitals and HCWs. Tools of this nature can be utilized by hospitals to explore aspects of preparedness and give insights to the knowledge, skills, and mental health of HCWs, as perceived by the HCW themselves. However, further exploration of the psychometric properties of the questionnaire is recommended to assess item performance and stability of the latent factors .

## Data Availability

The datasets generated during and analysed during the current study are not publicly available because some parts of the data can breach institutional privacy but are available from the corresponding author on reasonable request.

## Abbreviations

COVID-19: Coronavirus Disease 2019
HCWs: health care workers
MERS: Middle East Respiratory Syndrome
PTSD: post-traumatic stress disorder
SARS: Severe Acute Respiratory Syndrome
SARS-CoV-2: Severe Acute Respiratory Syndrome Coronavirus 2
SQIDORP: Staff Questionnaire for Infectious Disease Outbreak Readiness and Preparedness
